# Microscopic Peritoneal Residual Disease after Complete Macroscopic Cytoreductive Surgery for Advanced High Grade Serous Ovarian Cancer

**DOI:** 10.3390/jcm10010041

**Published:** 2020-12-25

**Authors:** Henri Azaïs, Anne-Sophie Vignion-Dewalle, Marine Carrier, Jeremy Augustin, Elisabeth Da Maïa, Alix Penel, Jérémie Belghiti, Marianne Nikpayam, Clémentine Gonthier, Laurine Ziane, Serge Mordon, Pierre Collinet, Geoffroy Canlorbe, Catherine Uzan

**Affiliations:** 1 Department of Gynecological and Breast Surgery and Oncology, Assistance Publique-Hôpitaux de Paris (AP-HP), Pitié-Salpêtrière University Hospital, 75013 Paris, France; marine.carrier@free.fr (M.C.); jeremie.belghiti@aphp.fr (J.B.); marianne.nikpayam@aphp.fr (M.N.); clementine.gonthier@aphp.fr (C.G.); geoffroy.canlorbe@aphp.fr (G.C.); catherine.uzan@aphp.fr (C.U.); 2 U1189-ONCO-THAI-Laser Assisted Therapy and Immunotherapies for On-cology, CHU Lille, Université de Lille, INSERM, F-59000 Lille, France; anne-sophie.vignion@inserm.fr (A.-S.V.-D.); laurine.ziane@inserm.fr (L.Z.); serge.mordon@inserm.fr (S.M.); pierre.collinet@chru-lille.fr (P.C.); 3 AP-HP, Pitié-Salpêtrière Hospital, Department of Pathology, 75013 Paris, France; jeremy.augustin@aphp.fr (J.A.); elisabeth.da-maia@aphp.fr (E.D.M.); 4 AP-HP, Pitié-Salpêtrière Hospital, Centre de Pharmaco-épidémiologie de l’APHP (CEPHEPI), 75013 Paris, France; Alix.penel@aphp.fr; 5 CHRU Lille, Jeanne de Flandre Hospital, Department of Gynecology, 59000 Lille, France; 6 Cancer Biology and Therapeutics, Centre de Recherche Saint-Antoine (CRSA), Sorbonne University, INSERM UMR_S_938, 75020 Paris, France; 7 Institut Universitaire de Cancérologie (IUC), Sorbonne University, 75013 Paris, France

**Keywords:** epithelial ovarian cancer, peritoneal carcinomatosis, cytoreductive surgery, gynecologic oncology

## Abstract

Background: Epithelial ovarian cancers (EOC) are usually diagnosed at an advanced stage and managed by complete macroscopic cytoreductive surgery (CRS) and systemic chemotherapy. Peritoneal recurrence occurs in 60% of patients and may be due to microscopic peritoneal metastases (mPM) which are neither eradicated by surgery nor controlled by systemic chemotherapy. The aim of this study was to assess and quantify the prevalence of residual mPM after complete macroscopic CRS in patients with advanced high-grade serous ovarian cancer (HGSOC). Methods: A prospective study conducted between 1 June 2018 and 10 July 2019 in a single referent center accredited by the European Society of Gynecological Oncology for advanced EOC management. Consecutive patients presenting with advanced HGSOC and eligible for complete macroscopic CRS were included. Up to 13 peritoneal biopsies were taken from macroscopically healthy peritoneum at the end of CRS and examined for the presence of mPM. A mathematical model was designed to determine the probability of presenting at least one mPM after CRS. Results: 26 patients were included and 26.9% presented mPM. There were no differences in characteristics between patients with or without identified mPM. After mathematical analysis, the probability that mPM remained after complete macroscopic CRS in patients with EOC was 98.14%. Conclusion: Microscopic PM is systematically present after complete macroscopic CRS for EOC and could be a relevant therapeutic target. Adjuvant locoregional strategies to conventional surgery may improve survival by achieving microscopic CRS.

## 1. Introduction

Epithelial ovarian cancer (EOC) is usually diagnosed at an advanced stage and the clinical situation is characterized by the presence of extensive peritoneal metastases (PM) within the abdominal cavity. Complete macroscopic cytoreductive surgery (CRS), which is the surgical removal of all peritoneal lesions suspected of cancer and visible to the surgeon, remains the cornerstone of the management of EOC, in combination with platinum-based chemotherapy [1,2,3]. The absence of residual disease after surgery has been shown to improve prognosis with a high level of evidence [4,5,6,7,8,9,10,11]. Nevertheless, despite improvement in surgical skills and adjuvant treatment strategies, peritoneal recurrence occurs in 60% of patients after primary clinical remission [12,13].

One of the mechanisms leading to peritoneal recurrence could be that microscopic clusters of cancerous cells that have neither been eradicated by surgery, nor controlled by systemic chemotherapy remain after CRS [14]. Particular attention thus needs to be paid to the improvement of locoregional treatment strategies in addition to conventional surgery, especially given that peritoneal progression is responsible for morbidity and complications leading to death in most cases.

Although therapeutic alternatives are based on microscopic peritoneal involvement in EOC to justify more intensive treatment of the peritoneum, there are very few studies in the literature investigating its detection and potential role in the progression of cancer.

The aim of this study was to assess and to quantify the prevalence of residual microscopic PM (mPM) after complete macroscopic CRS in patients with advanced high grade serous ovarian cancer (HGSOC) using a mathematical approach.

## 2. Methods

### 2.1. Design

This was a prospective single-center study conducted between 1 June 2018 and 10 July 2019 at the Department of Gynecological and Breast Surgery and Oncology, Pitié-Salpêtrière University Hospital (Paris, France). The objective was to assess and to quantify the prevalence of residual mPM after complete macroscopic CRS in patients with advanced high grade serous ovarian cancer.

### 2.2. Funding

The study was sponsored by Assistance Publique—Hôpitaux de Paris (Délégation à la Recherche Clinique et à l’Innovation). Award number: F101H1. Recipient: Henri Azaïs, M.D., Ph.D.

The funding made it possible to purchase the consumables necessary for the analysis of the biopsies as well as to finance the administrative costs incurred to submit the project and monitor it (recruitment of staff for data collection, etc.).

### 2.3. Patients

All women over 18 years old who presented advanced-stage HGSOC at our center during the study period were invited to participate if complete macroscopic CRS could be performed at primary or interval debulking surgery. Advanced stage was defined by a stage IIB-IV of the 2014 International Federation of Gynecology and Obstetrics (FIGO) classification.

The patients were treated in accordance with international recommendations after systematic pre-therapeutic validation during a multidisciplinary consultation. Our department of surgery is certified by the European Society of Gynecologic Oncology (ESGO) for the management of advanced stage EOC. An exploratory laparoscopy was performed to assess the resectability of the lesions. The Peritoneal Carcinomatosis Index (PCI) [15] and the laparoscopic Fagotti score [16] were used to quantify macroscopic peritoneal spread. Complete macroscopic CRS was performed by laparotomy at the same time as, or a few days after, laparoscopy. Macroscopic complete CRS included at least peritoneal cytology, total hysterectomy, bilateral salpingo-oophorectomy, infragastric omentectomy, appendectomy, and other surgical procedures allowing the removal of all visible suspicious peritoneal lesions. Pelvic and para-aortic lymphadenectomy were performed according to the recommendations [17] and the results of the LION study [18]. When there was no suspicion of radiological lymph node involvement during initial management or at palpation during surgery, we did not perform routine lymphadenectomy.

The following clinical and pathological variables were collected: age, comorbidities, parity, surgical procedure, stage according to the 2014 classification of the International Federation of Gynecology and Obstetrics (FIGO) [19], final pathological analysis, adjuvant therapies, *BRCA* status (in accordance with international guidelines, a *BRCA* mutation is systematically sought to adapt further management of patients), date of recurrence, death, or latest news.

### 2.4. Sampling Protocol and Pathological Analysis

Peritoneal biopsies were taken at the end of the CRS by a senior surgeon experienced in ovarian cancer surgery. The end of the CRS was defined by complete removal of macroscopic PM. Sampling was performed according to the following protocol: surgeons took peritoneal biopsies of approximately 4 cm^2^. One biopsy was taken per location designated in the PCI if this location was initially affected by macroscopic PM (maximum of 13 biopsies per operation). Peritoneal biopsies were taken from areas outside of those that might suggest to the surgeon a scar of peritoneal metastases that had responded to neoadjuvant chemotherapy.

These samples were sent to the pathology department for analysis. Hematein, eosin, saffron (HES) stained slides were read by both a specialized pathologist and a gynecologist to search the presence of mPM. Microscopic PM were defined by the observation of adenocarcinoma foci on the HES slide of a biopsy sample or by the expression of PAX8 by isolated clusters of tumor cells (immunohistochemistry). The results were noted in the pathological report.

### 2.5. Primary Endpoint

The primary endpoint was to assess the presence of PM on pathological examination of peritoneal biopsies taken from macroscopically healthy peritoneum (MHP) at the end of complete macroscopic CRS. The presence of such metastases defined the existence of mPM.

### 2.6. Statistical Analysis

Categorical variables were expressed as numbers (percentage). Continuous variables were expressed as means (±standard deviation, SD) or medians (range). Comparisons in patient characteristics between the two study groups (with mPM or without mPM) were completed using Student’s *t*-tests for continuous variables and the Mann–Whitney test for non-continuous variables. Statistical testing was completed at the two-tailed α level of 0.05. Data were managed with an Excel database (Microsoft Corporation, Redmond, WA, USA) and analyzed using R 3.5.1 software, available online (https://cran.r-project.org/).

### 2.7. Mathematical Analysis

We designed a mathematical model aiming to determine the probability of patients with EOC presenting at least one mPM which would have not been detected during complete macroscopic CRS. This model also aimed to determine the likelihood of the EOC having spread into the peritoneum. Only an outline of the model is provided in this section. A complete description of the model can be found in Supplementary Material 1.

The only information available to the model for achieving its aims was the patients’ biopsies results. In this regard, it is important to note that the model assumes the same number of biopsies for all patients. To include all the available patients’ biopsies results, this number was set to the maximum number of biopsies for all patients, which, as reported above, was 13. Biopsy data of patients therefore had to be completed accordingly: for patients with fewer than 13 biopsies, “artificial” biopsies without mPM were added to the “actual” peritoneal biopsies to obtain a total number of 13 biopsies.

Once this was done, the model was built based on the following four main parts.

The first part of the model involved estimating the probabilities that 0, 1, 2, …, or all the 13 biopsies have mPM. This estimation was performed from the “completed” biopsies data of patients. First, a relative histogram of these data was created with number of biopsies with mPM along the horizontal axis and percentage of patients (relative frequency) along the vertical axis. Second, according to the shape of this histogram, a common distribution was selected and adjusted to best fit the histogram and therefore to best estimate the required probabilities.

The second part of the model was to consider the peritoneal surface as a set of small sub-surfaces with area equal to that sampled by one biopsy, namely, approximately 4 cm^2^. The total number of these sub-surfaces was deduced as the ratio of the average peritoneal surface area in patients to 4 cm^2^. The peritoneal surface area of each patient had therefore to be computed. First, the body surface area was derived from the height and weight of the patient by applying a formula usually used in clinical practice. Second, based on the study by Albanese et al., this body surface area was used as an estimate of the peritoneal surface area of the patient [20].

The third and key part of the model focused on the determination of the probability of patients with EOC presenting at least one mPM, which would have not been detected during complete macroscopic CRS. With the above-introduced division of the peritoneal surface, this probability was regarded as the probability that at least one sub-surface has an mPM. Following Equation (S2) in Supplementary Material 1, this latter can be directly derived from the probabilities that 0, 1, 2, …, or all the sub-surfaces have an mPM. These probabilities therefore had to be estimated. This estimation was performed involving the probabilities resulting from the first part of the model (i.e., the probabilities that 0, 1, 2, …, or all the 13 biopsies have an mPM), the constrained least-squares optimization method and probabilistic principles such as conditional probability, hypergeometric distribution, and law of total probability (Equation (S11), Supplementary Material 1).

Due to the previous completion of the biopsies data of patients, which limits the occurrence of biopsies with mPM, the probability of patients with EOC presenting at least one mPM was probably underestimated. The conclusions, which could be drawn in case of a significant value for the probability of patients with EOC presenting at least one mPM, would therefore be further strengthened if no data completion was required.

The fourth and last part of the model consisted of quantifying the likelihood of the EOC having spread into the peritoneum. From the probabilities estimated in the third part of the model (i.e., the probabilities that 0, 1, 2, …, or all the sub-surfaces has an mPM), the number *n*—such that the probability that more than *n* of the sub-surfaces has an mPM is equal to 5% (25%, 50%, 75% and 95%)—was deduced (Equation (S3) in Supplementary Material 1). These numbers, which can be considered as a kind of percentile, provide the simplest indicators of the degree of spread of EOC into the peritoneum. In fact, the higher these numbers are, the greater the spread of EOC within the peritoneum is.

All the computations were performed using the Matlab software (The MathWorks, Natick, MA, USA).

### 2.8. Ethics

The research protocol was approved by a national Ethics Committee (PPC Sud Est II, ID-RCB 2017-03431-52; Date of approval: 16/05/2018) and registered on clinicaltrials.gov (NCT03754569). The collection of patient data was declared to the French “Commission Nationale Informatique et Liberté” (CNIL-Q9d2264273M). Each patient signed a written informed consent after receiving clear and detailed information about the research protocol.

In the absence of preliminary data and given the possibility of not detecting mPM at the end of the complete CRS, it did not seem legitimate to subject many patients to an additional surgical procedure. We therefore made the arbitrary choice of conducting an initial exploratory prospective pilot study based on a cohort of consecutive patients over one year.

## 3. Results

### 3.1. Population Characteristics

Twenty-six patients with advanced HGSOC were included after complete macroscopic CRS. Among them, seven (26.9%) presented with mPM. The characteristics of the entire population, and those of the two subgroups (without or with mPM), are presented in Table 1.

The mean age of the patients overall at the time of diagnosis was 65.3 years ± 11.1. Three patients (11.5%) had a somatic *BRCA1* mutation, one (3.8%) had a *BRCA2* mutation, and 18 (69.2%) had no mutation. For the remaining four patients, analysis was ongoing at the time of writing this manuscript.

All the patients received chemotherapy with carboplatin and paclitaxel. Chemotherapy was administered in a neoadjuvant setting (NACT) for 23 patients (88.5%). The median follow-up time was 492 (262–862) days. During this period, eight (30.8%) recurrences occurred with a median time of 356 (213–862) days, six (31.6%) in the group without mPM and two (28.6%) in the group with mPM. Three patients died during the study period.

There were no statistical differences between the two groups regarding PCI at diagnosis (*p* = 0.16) or at the time of CRS (*p* = 0.40), the Fagotti score at diagnosis (*p* = 0.26) or at the time of CRS (*p* = 0.97), or the CA 125 levels at diagnosis (*p* = 0.57) or at the time of CRS (*p* = 0.30), although these parameters were still higher at diagnosis for the patients with identified mPM.

The number of courses of NACT did not differ in patients with or without mPM (*p* = 0.84).

### 3.2. Analysis of Peritoneal Biopsies in Macroscopically Healthy Peritoneum

A median of 7 [3,4,5,6,7,8,9,10,11,12,13] MHP biopsies were taken per patient with no difference between the two groups: 6 [3,4,5,6,7,8,9,10,11,12] for patients with mPM and 7 [3,4,5,6,7,8,9,10,11,12,13] for patients without (*p* = 0.41).

**Figure 1:**
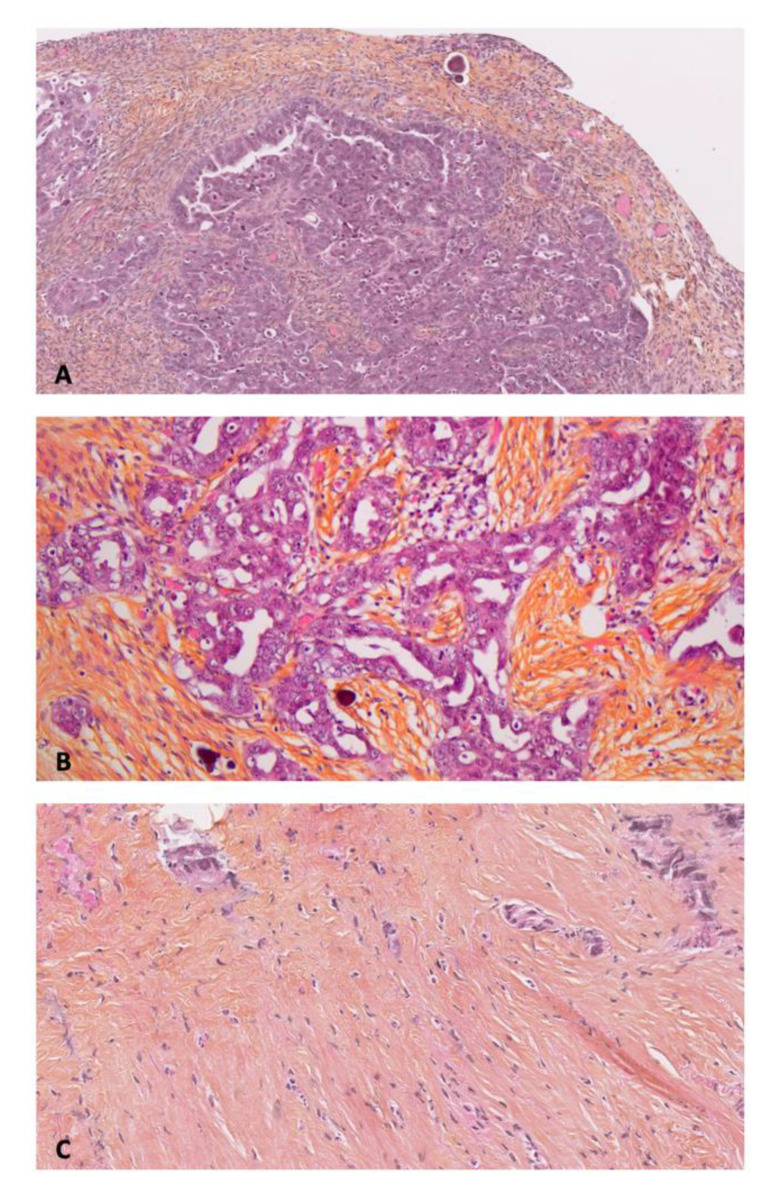
Anatomopathological examination of primary and peritoneal lesions of high grade serous ovarian carcinoma (HES: Hematein, Eosin, Saffron). (**A**) Primary cancer × 10. (**B**) Macroscopic peritoneal metastase × 20. (**C**) Microscopic peritoneal metastase × 10.

Microscopic PM (Figure 1) were located in the Morrison space (*n* = 2), small omentum (*n* = 2), right diaphragmatic peritoneum (*n* = 2), pelvic peritoneum (*n* = 1), parieto-colic gutter (*n* = 1), mesorectal peritoneum (*n* = 1), mesenteric peritoneum, and prevesical peritoneum (*n* = 1).

### 3.3. Mathematical Analysis

**Figure 2:**
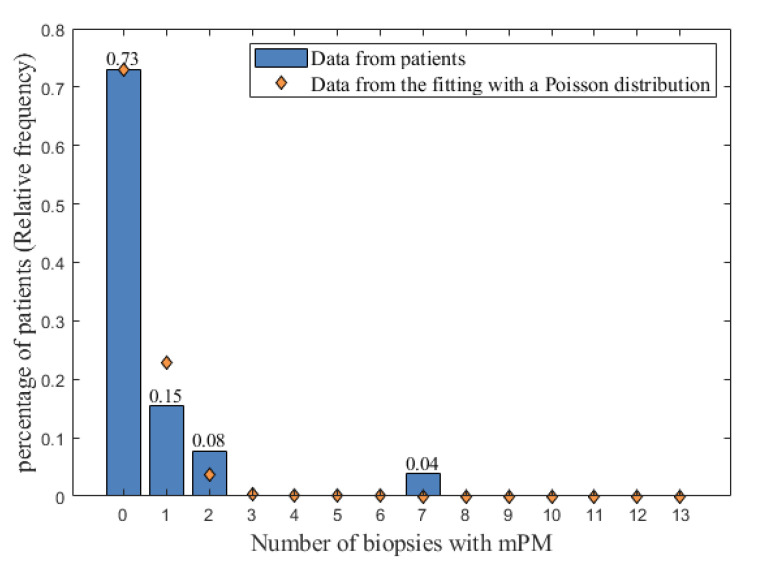
The relative histogram of the number of biopsies with mPM (blue bars) fitted with a Poisson distribution (orange diamonds). The possible values for the number of biopsies with mPM are reported on the x-axis, and the relative frequency of occurrences of these possible values are represented by blue bars on the y-axis. mPM: microscopic peritoneal metastases.

Of the 26 patients, each associated with 13 biopsies (after data completion as described above), one patient had seven biopsies with mPM, two had two biopsies with mPM and four had one biopsy with mPM. For the remaining 19 patients, mPM was not detected in any of the 13 biopsies. As stated in the first part of the model, these results were displayed as a relative histogram (Figure 2). From this histogram, results were found to be properly described by a Poisson distribution. An optimization was therefore performed using the Matlab software to determine the Poisson distribution, which best fits the relative histogram. The Poisson distribution obtained by fitting (orange diamonds in Figure 2) was used to assign the probabilities that 0, 1, 2, …, or all the 13 biopsies have mPM.

The peritoneal surface areas of the 26 patients were computed as described in the second part of the model and the average value was found to be approximately equal to 16,000 cm^2^ (minimum, 14,000 cm^2^; maximum, 20,000 cm^2^). From this value, the total number of the sub-surfaces required to describe the peritoneal surface was deduced to be 4000.

According to the third part of the model, the probabilities that 0, 1, 2, …, or all the 4000 sub-surfaces has an mPM were then estimated.

**Figure 3:**
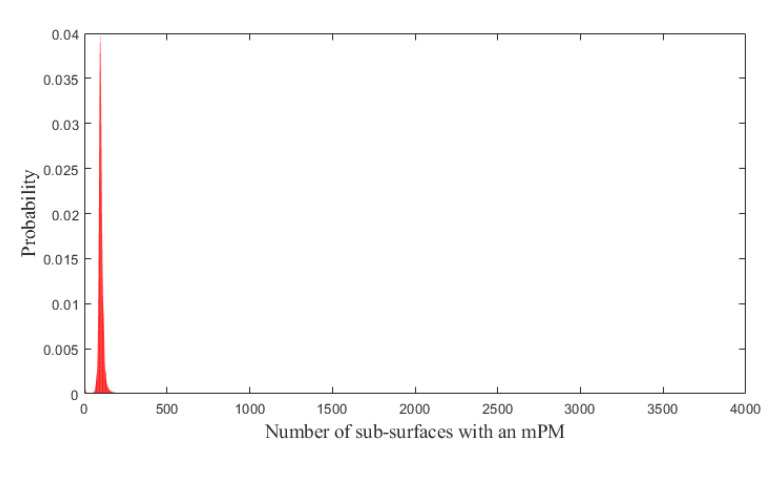

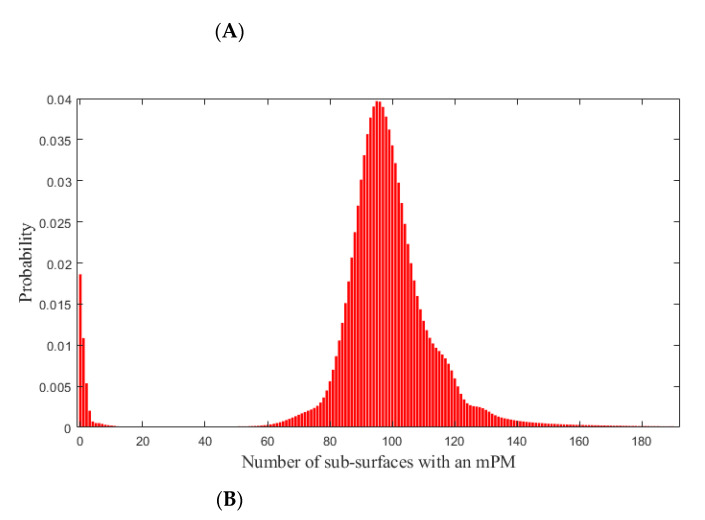
Probabilities that 0, 1, 2, …, or all the 4000 sub-surfaces of the peritoneal surface has an mPM (red bars). (**A**): Overview. (**B**): Zoom on the range (0; 200) in which the probabilities are maximal. mPM: microscopic Peritoneal Metastases.

Based on these probabilities shown in Figure 3, the probability of patients with EOC presenting at least one mPM, which would have not been detected during complete macroscopic CRS was computed to be 98.14%. This value shows that it is likely that residual mPM are systematic after complete macroscopic CRS.

The indicators of the degree of spread of EOC into the peritoneum, defined in the fourth part of the model, were also computed (Table 2).

From Table 2 (last column), the probability of observing more than 69 sub-surfaces with mPM (this corresponds to 69/4000 ≈ 1.7% of the peritoneal surface area) is about 95%. The probability that more than 2.62% of the peritoneal surface area (105 areas of the 4000) has mPM was found to be approximately 25% (third column).

## 4. Discussion

According to our findings, the probability that microscopic residual disease is still present after complete macroscopic CRS in patients with advanced stage HGSOC is 98.14%. Moreover, our results suggest that 95% of patients have more than 69 residual locations with mPM after complete macroscopic CRS (69/4000 ≈ 1.7% of the peritoneal surface area), which highlights the degree of microscopical spread of EOC into the peritoneum. These results strongly suggest that adjuvant locoregional therapies should be explored in this setting since the presence of mPM could explain the high rate of peritoneal recurrence. Microscopic PM are often mentioned in the literature and their existence supports the use of additional locoregional therapeutic strategies to surgery. However, the demonstration that residual mPM are systematic after surgery had never been established before. The mathematical model used in this study is essential to extrapolate to all patients an observation that is difficult to obtain in each of them given the non-visible nature of the metastases sought over an area as large as that of the peritoneum.

The few publications reporting pathological evidence of mPM are based on studies evaluating the accuracy of systematic peritoneal staging in presumed early-stage EOC. Among these, in a study assessing the value of systematic peritoneal biopsy and omentectomy in early-stage EOC, Shroff et al. found mPM in the MHP of 5% of patients [21]. According to Ayhan et al., 31.4% of patients with presumed early-stage EOC were upstaged after occult metastases were identified on final pathologic examination. These metastases were located on isolated positive peritoneal cytology in 35.8%, on the Supplementary Material 1 in 7.5%, and were peritoneal or omental metastases in 15.1% of the cases (3.8% and 11.3%, respectively). Five-year survival was 61·8% in the patients presenting with occult metastases, and 88.25% in the others. Prognosis was poorer if peritoneal or omental metastases were detected [22]. In a study by Soper et al., aiming to determine the yield of comprehensive restaging laparotomy in 30 women with presumed early-stage EOC who had undergone incomplete initial staging procedures, two-thirds of the upstaging resulted from positive cytology, biopsies of adhesions, or random peritoneal biopsies [23]. Other series have reported mPM in systematic peritoneal biopsies in early-stage EOC in 1.2% to 9.3% of cases [22,23,24,25]. Gadducci et al. retrospectively studied the presence of mPM in advanced stage EOC during second-look surgery, after CRS and adjuvant chemotherapy. Microscopic residual disease was found on peritoneal biopsies or peritoneal cytology in 79 of the 95 patients (83.2%) [26]. To our knowledge, no previous study has been conducted to assess the prevalence of mPM at the time of CRS for advanced stage EOC. The size of this pilot study is limited, but enough to objectively confirm a hypothesis that is widely spread but never proven. For this reason, and in view of the methodology involving additional surgical specimens (peritoneal biopsies) potentially causing complications, we have decided not to include more patients in this study.

We found mPM on MHP in seven of 26 our prospectively included patients (26.9%). Biopsies represent a negligible sampling of the whole peritoneal surface. The prevalence of mPM is therefore higher than that estimated by systematic sampling hence the interest of mathematical modeling. The model we applied demonstrated that mPM are present in nearly 100% of patients.

The peritoneum is the first site of recurrence in EOC [27] and responsible for morbidity and mortality. The 2-years peritoneal recurrence rate is 20% for early stages I to IIA and 62.1% for advanced stages IIB to IV [28] with recurrences occurring on treated peritoneum or peritoneum described as initially healthy during CRS. Our data support the hypothesis that microscopic clusters of cancerous cells that are not eradicated by surgery, nor controlled by systemic chemotherapy, and may be one of the mechanisms leading to peritoneal recurrence [14] as most of the patients underwent surgery after neoadjuvant chemotherapy.

No surgical parameters such as PCI or Fagotti score values were associated with the identification of mPM in our series. The biologic markers (CA 125) were not statistically different between patients with evidence of mPM and those without proven microscopic involvement. Nevertheless, there was a trend towards higher values of these surgical and biological parameters in the patients with mPM. These results emphasize the fact that a higher spread of macroscopic peritoneal involvement is naturally associated with a higher probability of detecting mPM on systematic biopsy on MHP. On the other hand, there was no statistical differences between the two groups, which allowed us to integrate all the patients in the same mathematical model.

It has been clearly established that complete surgical removal of macroscopic lesions improves overall survival (OS) and recurrence-free survival (RFS). In the Cochrane Review published by Elattar et al., analyses showed that in the groups of women with EOC in whom complete macroscopic CRS could be achieved, OS and RFS were significantly prolonged [11]. The correlation between increased survival and the quality of the macroscopic CRS raise the question of the potential effect of achieving a microscopic cytoreduction on prognosis.

This study thus reinforces the importance of developing new ways to identify and remove more PM and to treat the remaining peritoneum. Fluorescence-guided surgery may be helpful for identifying mPM during CRS [29]. Using a fluorescent dye, a specific fluorescence emitted by tumor tissue can be detected thereby mapping the peritoneal lesions and guiding the surgeon in resecting all visible lesions, including smaller ones. Van Dam et al. published the first in-human use of a tumor-specific fluorescence imaging technique in EOC, combining fluorescein-isothiocyanate with folate (EC17) thus targeting overexpressed folate receptor (FR) cells [30]. There was an excellent correlation between fluorescent tissue (expressing FR) and non-fluorescent tissue (non-malignant tissue, or in one case, malignant tissue not expressing FR). No fluorescence was detected in healthy tissues. Fluorescence from tumor tissue was observed from 2 to 8 h after drug administration [30]. With the same compound, Tummers et al. reported that 16% of histologically proven PM detected with fluorescence were not visible under white light [31]. A study performed with another folate-targeted fluorescent agent (OTL38) showed that it was possible to detect 29% more PM than under white light exposure [32]. In a phase I clinical trial performed by Liu et al., specificity for the detection of PM of EOC under blue light during fluorescence-guided CRS was 100%, and the surgery was combined with hyperthermic intraperitoneal chemotherapy (HIPEC) [33]. Nevertheless, fluorescence-guided surgery is still limited by the accuracy of the optical systems [29], and no studies have demonstrated the benefit of fluorescence-guided surgery in terms of survival in patients with EOC.

The goal of intraperitoneal (IP) chemotherapy is to effectively treat the residual PM following CRS by administrating cytotoxic agents directly into the peritoneal cavity [13]. Indeed, the IP route has the advantage of bypassing the poor vascularization of small volume disease and to increase drug concentrations in these tumors. IP administration allows cisplatin to penetrate tumors to a maximum depth of 1–3 mm [13]. The amount of drug delivered by this route seems to be twice the maximally tolerated dose of cisplatin delivered intravenously [34]. Chemotherapy can be administered at an elevated temperature of 42 degrees (HIPEC), which increases the cytotoxic effect and the penetration of the product into the tissues [35]. The latest French recommendations published in 2018 specify that HIPEC may be proposed during interval surgery with a residue of less than 10 mm in patients with an initially unresectable stage FIGO III primary ovarian, tubal or peritoneal carcinosis after three courses of intravenous chemotherapy [36]. These recommendations follow findings by van Driel et al. who showed an improvement in survival without any increase in side effects with HIPEC treatment of stage III EOC during interval CRS [27]. According to Gourley et al., the benefits of IP chemotherapy in terms of outcome is high [37]. However, HIPEC is not considered as a standard of care in the latest 2019 recommendations of the European Society of Gynecologic Oncology (ESGO) for the management of EOC [38]. Large, international, prospective studies are needed to clearly quantify the efficacy of IP chemotherapy as well as to determine accurate indications and optimal drug regimen for a maximal benefit for survival of patients.

Photodynamic therapy (PDT) is undergoing preclinical evaluation and could be of interest for the treatment of mPM. PDT is based on the administration of a photosensitizer that accumulates in cancer cells. Intraoperative illumination at an appropriate wavelength results in activation of the photosensitizer which is responsible for a photobiological reaction leading to the formation of cytotoxic reactive oxygen species. This results in the death of the tumor cells and a potential abscopal effect mediated by the release of extracellular vesicles with immune-activating properties [14,39]. Its double selectivity (photosensitizer specificity for the tumor and activation controlled by illumination) means there are no distant systemic effects which represents a theoretical advantage over HIPEC. A folate-coupled photosensitizer [40] could provide sufficient specificity to warrant evaluation by a clinical trial.

Our results demonstrate that conventional CRS alone cannot ensure cytoreduction of the entire peritoneum. The significance of mPM after complete macroscopic CRS is different between patients who received NACT or not before CRS. The use of NACT is a growing trend in the management of advanced HGSOC, with equivalent oncologic outcome in comparison with primary CRS when complete macroscopic resection has been achieved. The proportion of patients operated in primary CRS is low in our cohort, allowing to extrapolate our results mainly for the patient operated after NACT, even if the peritoneal spread after NACT is probably a reflection of the initial spread of the disease. The quality of CRS remains a decisive element in the management of patients. The presence of mPM after NACT could be related to visible metastases prior to chemotherapy, as suggested by the publication of Tate et al. [41], encouraging the surgeon to note locations of metastases at the initial exploratory laparoscopy to resect these locations in case of doubt at the time of interval CRS. Given the limitations of this study, which concerns a cohort with a limited number of patients (*n* = 26), the absence of a separate analysis of patients who have or not received NACT and the mathematical method that involves the use of hypotheses that induce limits in its interpretation, other studies would be expected to reinforce our findings. Further studies would also aim to clarify the role of mPM in the occurrence of peritoneal recurrence (especially since we do not have a long-term follow-up in our protocol), their prognostic impact, and to evaluate and develop new diagnostic and therapeutic techniques.

## 5. Conclusions

Patients with advanced EOC present with systematic mPM after complete macroscopic CRS. Even in the era of poly-ADP-ribose polymerase (PARP) inhibitors [42] and pharmaceutical developments that are expected to improve prognosis in this population, surgery will remain central and we must continually strive to improve the relevance and quality of our procedures.

Microscopic PM could be a therapeutic target that could improve survival in patients with EOC by decreasing the rate of peritoneal recurrence: resistance to conventional therapy and the high recurrence rate is likely due to residual disease. Given the prognostic importance of achieving complete macroscopic CRS, additional locoregional strategies to conventional surgery, such as fluorescence-guided surgery, HIPEC or PDT are expected to achieve microscopic CRS and thus improve both RFS and OS. Further studies are needed to determine the precise role of mPM on the occurrence of peritoneal recurrence or on chemotherapy resistance mechanisms, as well as their impact on prognosis.

## Figures and Tables

**Table 1:** Population characteristics.

	Overall Population	Without mPM	With mPM	*p*
	*n* = 26	*n* = 19	*n* = 7	
Age (mean ± SD)~years	65.3 ± 11.1	63.6 ± 11.6	69.9 ± 9.1	0.17
BMI (mean ± SD)~kg/m^2^	22.9 ± 3.7	23.2 ± 3.8	22.3 ± 3.5	0.64
Body surface (mean ± SD)~m^2^	1.65 ± 0.13	1.65 ± 0.15	1.62 ± 0.04	0.91
*BRCA* status				
*BRCA1*~*n* (%)	3 (11.5)	3 (15.8)	0 (0)	NA
*BRCA2*~*n* (%)	1 (3.8)	1 (5.3)	0 (0)	NA
No mutation~*n* (%)	18 (69.2)	12 (63.2)	6 (8.7)	NA
Unknown~*n* (%)	4 (15.4)	3 (15.8)	1 (14.3)	NA
Follow-up (after CRS), median (range)~days	492 (262–862)	495 (301–862)	376 (262–714)	0.27
Recurrence~*n* (%)	8 (30.8)	6 (31.6)	2 (28.6)	NA
RFS (median (range))~days	356 (213–862)	359 (292–862)	307 (213–400)	NA
Death~*n* (%)	3 (11.5)	2 (10.5)	1 (14.3)	NA
Peritoneal metastases spread at diagnosis				
PCI (median (range))~/39	13 (3–31)	11 (3–31)	21·5 (10–31)	0.16
Fagotti score (median (range))~/14	6 (0–12)	4 (0–10)	8 (6–12)	0.26
CA 125 (median (range))~UI/mL	590 (19–8000)	579 (19–4042)	600 (40–8000)	0.57
Peritoneal metastases spread at CRS				
PCI (median (range))~/39	11 (0–20)	11 (0–20)	4 (1–15)	0.4
Fagotti score (median (range))~/14	4 (0–8)	4 (0–8)	2 (2–8)	0.97
CA 125 (median (range))~UI/mL	20 (10–1162)	20 (10–1162)	253 (11–904)	0.3
FIGO Stage				
IIIC~*n* (%)	18 (69.2)	13 (68.4)	5 (71.4)	NA
IV~*n* (%)	8 (30.8)	6 (31.6)	2 (28.6)	NA
Number of biopsies (MHP) (median (range))	7 (3–13)	7 (3–13)	6 (3–12)	0.41
~*n* (%)	23 (88.5)	16 (84.2)	7 (100)	NA
Number of courses before CRS (median (range))	5 (3–9)	6 (3–9)	4 (3–9)	0.84

BMI (Body Mass Index); CRS (Cytoreductive Surgery); RFS (Recurrence-Free Survival); PCI (Peritoneal Cancer Index); MHP (Macroscopically Healthy Peritoneum); NACT (Neoadjuvant Chemotherapy).

**Table 2:** Numbers *n* (2nd row) so that the probability to have more than *n* sub-surfaces with mPM is equal to specific percentages (1st row). mPM: microscopic Peritoneal Metastases.

Probability to Have More Than *n* Possible Areas of Biopsy with Microscopic Metastases	5%	25%	50%	75%	95%
*n*	125	105	97	91	69

## Data Availability

The data presented in this study are available on request from the corresponding author.

## Supplementary Materials

The following are available online at https://www.mdpi.com/2077-0383/10/1/41/s1, Supplementary Material 1: Mathematical method.
