# Supplementary material for: Microscopic Peritoneal Residual Disease after Complete Macroscopic Cytoreductive Surgery for Advanced High Grade Serous Ovarian Cancer
Source: J Clin Med. 2020 Dec 25;10(1):41. doi: 10.3390/jcm10010041 (PMC7795826; doi:10.3390/jcm10010041)
Supplement: Supplementary file 1 [file jcm-10-00041-s001.pdf]

Supplementary Material 1. Mathematical method.

Let p_P be the number of patients included in the study.

Let m_B be the number of peritoneal biopsies, which have been carried out in the peritoneum of each patient to histologically determine if microscopic peritoneal metastases (mPM) of epithelial ovarian cancer (EOC) are present (all patients were assumed to have undergone the same number of biopsies). We assumed 100% reliability for biopsy (i.e., no false positives and no false negatives).

Let B^+ be the discrete random variable that gives the number of biopsies with mPM in each patient. Given the above definition of m_B, the possible values for B^+ are 0, 1, 2, …, m_B (note, that uppercase letters are used for random variables, whereas numbers and lowercase letters are related to the possible values of these random variables). Let $B_m^+ = \{m\}$ be the event corresponding to the occurrence of m biopsies with mPM among the m_B performed ones. The probability associated with the event B_m^+ was denoted by $P(B_m^+)$. All the probabilities $P(B_0^+)$, $P(B_1^+)$, … and $P(B_{m_B}^+)$ were assumed to have been estimated from the patient data and therefore to be known.

Let S_P (cm[2]) be the average peritoneal surface area in adults.

If we consider that each biopsy samples a surface area of S_B (cm[2]), a total of $n_A = \frac{S_P}{S_B}$ peritoneal biopsies should have been carried out in each patient to assess the entire peritoneal surface. The peritoneum in each patient can therefore be described as the union of n_A possible areas of biopsy. Of course, n_A is far higher than m_B $(n_A \gg m_B)$.

Let A^+ be the discrete random variable that gives the number of these possible areas of biopsy that actually have mPM in each patient. A^+ can then have the values 0, 1, 2, up to n_A. We denoted by $A_n^+ = \{n\}$ the event that n of the n_A possible areas of biopsy actually have mPM and by $P(A_n^+)$ the probability for this event to occur. The events A_0^+, A_1^+, …, $A_{n_A}^+$, which are mutually exclusive (both events cannot occur at the same time) and together cover all possible values of A^+, form a partition. As the result, the sum of the probabilities of these $(n_A + 1)$ events is equal to 1 (Equation (S1)).

$$\sum_{n=0}^{n_A} P(A_n^+) = 1 \qquad \text{(S1)}$$

The probabilities $P(A_0^+)$, $P(A_1^+)$, … and $P(A_{n_A}^+)$ might be very useful for optimizing treatment for patients. The deduction from $P(A_0^+)$, $P(A_1^+)$, … and $P(A_{n_A}^+)$, is the probability of the event that at least one of the n_A possible areas of biopsy has mPM. This event, above referred to as $A_{?a?1}^+$, can be written as the union of the events A_1^+, A_2^+, …, $A_{n_A}^+$, so that its probability can be determined as follows:

$$P(A_{\geq 1}^+) = P(A_1^+ \cup A_2^+ \cup \ldots \cup A_{n_A}^+) = P(A_1^+) + P(A_2^+) + \cdots + P(A_{n_A}^+) =_{Eq(1)} 1 - P(A_0^+) \qquad \text{(S2)}$$

where $\cup$ represents the union operator and the two last equalities result from the fact that the events A_0^+, A_1^+, …, $A_{n_A}^+$ form a partition.

In this paper, we were particularly interested in this probability: the higher it is, the more likely the peritoneum is affected by mPM and the more beneficial the use of an adjuvant treatment to cytoreductive surgery.

Another information, the probabilities $P(A_0^+)$, $P(A_1^+)$, … and $P(A_{n_A}^+)$ would provide, relates to the spread of EOC within the peritoneum. Let n_P be the integer between 0 and n_A such that the probability of the event that more than n_P possible areas of biopsy have mPM is about P (Equation (S3)).

$$n_P \in \{0, 1, \ldots, n_A\} \;\middle|\; P(A_{\geq n_P}^+) = P(A_{n_P}^+ \cup A_{n_P+1}^+ \cup \ldots \cup A_{n_A}^+) = \sum_{n=n_P}^{n_A} P(A_n^+) \approx P \qquad \text{(S3)}$$

where $A_{?a?n_P}^+$ denotes the event that more than n_P possible areas of biopsy have mPM and the equalities result from the fact that the events A_0^+, A_1^+, …, $A_{n_A}^+$ form a partition.

The values of P we were interested in here were the standard 5%, 25%, 50%, 75% and 95%. The corresponding integers n_P can be considered as kind of percentiles. Take for example $P = 25\%$: $n_{25\%}$ is such that the probability to have more than $n_{25\%}$ possible areas of biopsy with mPM is equal to 25%; this implies that the probability of having less than $n_{25\%}$ possible areas of biopsy with mPM is equal to 75% and therefore $n_{25\%}$, can be viewed as the 75th percentile.

The higher the integers n_P are, the greater the spread of EOC within the peritoneum.

Unfortunately, the probabilities $P(A_0^+)$, $P(A_1^+)$, … and $P(A_{n_A}^+)$ were unknown. The major aim of our analysis was therefore to determine these probabilities from the little information we had and consisting of the probabilities $P(B_0^+)$, $P(B_1^+)$, … and $P(B_{m_B}^+)$.

To achieve this, we used the law of total probability. By applying this law with the above-defined partition A_0^+, A_1^+, …, $A_{n_A}^+$, the probability $P(B_m^+)$ and the set of probabilities $P(A_0^+)$, $P(A_1^+)$, … and $P(A_{n_A}^+)$ are linked to each other as follows (Equation (S4)).

$$P(B_m^+) = \sum_{n=0}^{n_A} P(B_m^+|A_n^+) \times P(A_n^+) \tag{S4}$$

With $P(B_m^+|A_n^+)$ is the conditional probability of B_m^+ given A_n^+, that is, the probability that B_m^+ occurs given that A_n^+ occurs (in other words, this is the probability that m of the m_B peritoneal biopsies present mPM given that n of the n_A possible areas of biopsy have mPM).

This conditional probability clearly follows a hypergeometric distribution. A hypergeometric distribution allows to describe the probability of obtaining a certain number of successes (here, biopsies with mPM, m) in a certain number of draws (here, biopsies, m_B) given a population of a certain size (here, possible areas of biopsy, n_A) containing a certain number of successes (here, possible areas of biopsy with mPM, n), without replacement. According to the definition of the hypergeometric distribution, $P(B_m^+|A_n^+)$ can therefore be expressed as in Equation (S5):

$$P(B_m^+|A_n^+) = \begin{cases} \dfrac{\binom{n}{m} \times \binom{n_A - n}{m_B - m}}{\binom{n_A}{m_B}} & \text{if } max(0, m_B + n - n_A) \le m \le min(n, m_B) \\ 0 & \text{otherwise} \end{cases} \tag{S5}$$

where the binomial coefficient $\binom{p}{k}$ is the total number of subsets of k distinct elements in a set of p elements, for any integers k and p such that $0 \le k \le p$.

Applying Equation (S4) for m between 0 and m_B, the following system of m_B linear equations in n_A unknowns were constructed (Equation (S6)):

$$\begin{pmatrix} P(B_0^+) \\ \vdots \\ P(B_{m_B}^+) \end{pmatrix} = \begin{pmatrix} P(B_0^+|A_0^+) & \dots & P(B_0^+|A_{n_A}^+) \\ \vdots & \ddots & \vdots \\ P(B_{m_B}^+|A_0^+) & \dots & P(B_{m_B}^+|A_{n_A}^+) \end{pmatrix} \begin{pmatrix} P(A_0^+) \\ \vdots \\ P(A_{n_A}^+) \end{pmatrix} \tag{S6}$$

In this system, only the probabilities $P(A_0^+)$, $P(A_1^+)$, … and $P(A_{n_A}^+)$ were unknown. To determine them, this system had to be solved. With many more unknowns than equations, this consistent system has infinite solutions (consistency was ensured by the above definition of $P(B_m^+|A_n^+)$ (Equation (S5)).

However, many of these solutions were not relevant from a probabilistic perspective. Bearing in mind that the solution consists of the probabilities of a partition, the solution had to meet two specific constraints: the first one, expressed in Equation (S1), states that the sum of $P(A_0^+)$, $P(A_1^+)$, … and $P(A_{n_A}^+)$ is equal to 1 and the second one indicates that $P(A_0^+)$, $P(A_1^+)$, … and $P(A_{n_A}^+)$ each lies within the range $[0; 1]$ (Equation (S7)):

$$0 \le P(A_n^+) \le 1 \text{ for any } n \text{ between 0 and } n_A \tag{S7}$$

with such constraints, the system in Equation (S6) could not be solved exactly. Instead, an approximation to the true solution could be obtained by the least squares method. The resulting solution would be the set of probabilities $P(A_0^+)$, $P(A_1^+)$, … and $P(A_{n_A}^+)$, which minimizes the fitting error defined in Equation (S8) while respecting the constraints of Equations (S1) and (S7).

$$E_{fitting} = \left\| \begin{pmatrix} P(B_0^+) \\ \vdots \\ P(B_{m_B}^+) \end{pmatrix} - \begin{pmatrix} P(B_0^+|A_0^+) & \dots & P(B_0^+|A_{n_A}^+) \\ \vdots & \ddots & \vdots \\ P(B_{m_B}^+|A_0^+) & \dots & P(B_{m_B}^+|A_{n_A}^+) \end{pmatrix} \begin{pmatrix} P(A_0^+) \\ \vdots \\ P(A_{n_A}^+) \end{pmatrix} \right\|^2 \tag{S8}$$

However, without additional constraint, particularly on the trend, this solution might contain irregularities. This means that there might be jumps between sequential probabilities: the probability that n of the n_A possible areas of

biopsy actually have mPM might, in fact, differ significantly from the probability that $(n+1)$ or $(n-1)$ of the n_A possible areas of biopsy actually have mPM. Such jumps would be obviously unphysical. This is why we introduced a smoothness constraint designed to discard solutions containing irregularities. The smoothness term T_{smooth}, we used to impose this smoothness constraint on the solution, involves the third-order difference as shown in Equation (S9) (the forward finite difference and the backward finite difference were applied for $n = 0$ and 1 and for $n = n_A - 1$ and n_A, respectively. The central finite difference was used for all the other n):

$$
\begin{aligned}
T_{smooth} &= [-P(A_0^+) + 3P(A_1^+) - 3P(A_2^+) + P(A_3^+)]^2 \\
&\quad + [-P(A_1^+) + 3P(A_2^+) - 3P(A_3^+) + P(A_4^+)]^2 \\
&\quad + \sum_{n=2}^{n_A-2} [-0.5\, P(A_{n-2}^+) + P(A_{n-1}^+) - P(A_{n+1}^+) + 0.5\, P(A_{n+2}^+)]^2 \\
&\quad + \left[-P\left(A_{n_{A-4}}^+\right) + 3P\left(A_{n_{A-3}}^+\right) - 3P\left(A_{n_{A-2}}^+\right) + P\left(A_{n_{A-1}}^+\right)\right]^2 \\
&\quad + \left[-P\left(A_{n_A-3}^+\right) + 3P\left(A_{n_{A-2}}^+\right) - 3P\left(A_{n_{A-1}}^+\right) + P\left(A_{n_A}^+\right)\right]^2 \\
&= \left\| \begin{pmatrix} -1 & +3 & -3 & +1 & 0 & & \\ 0 & -1 & +3 & -3 & +1 & & \\ -0.5 & +1 & 0 & -1 & +0.5 & & \\ & \ddots & \ddots & \ddots & \ddots & \ddots & \\ & & -0.5 & +1 & 0 & -1 & +0.5 \\ & & -1 & +3 & -3 & +1 & 0 \\ & & 0 & -1 & +3 & -3 & +1 \end{pmatrix} \begin{pmatrix} P(A_0^+) \\ P(A_1^+) \\ P(A_2^+) \\ P(A_3^+) \\ P(A_4^+) \\ \vdots \\ P\left(A_{n_A}^+\right) \end{pmatrix} \right\|^2
\end{aligned}
\tag{S9}
$$

The unspecified elements in matrix are equal to 0.

The closer to 0 this smoothness term is, the smoother the trend of the solution. Note that this smoothness term was designed for constant, linear, and quadratic solutions (the third-order difference and thus the smoothness term for such solutions indeed is zero).

This smoothness term was associated with the above defined fitting error (Equation (S8)) into a total error, which therefore accounts for both the compliance with smoothness and the closeness to the data (Equation (S10)):

$$E_{total} = E_{fitting} + ?\, a\, T_{smooth} \tag{S10}$$

with the parameter $?\,a$ controlling the trade-off between the compliance with smoothness and the closeness to the data: for large $?\,a$, the solution, resulting from the minimisation of the total error E_{total}, will be smoother at the expense of being further from the data, whereas for small $?\,a$, the solution will be closer to the data but with more irregularities.

The optimal value for $?\,a$ was determined using the cross-validation method [1].

The total error can be rearranged by using a matrix format so that the least squares method can be applied (Equation (S11)):

Applying the least squares method to minimize the total error under constraints of Equations (S1) and (S7) would provide the most suitable solution $P(A_0^+)$, $P(A_1^+)$, … and $P\left(A_{n_A}^+\right)$ to our problem.

This minimization was performed using the function "lsqlin" of the Matlab software.

Reference

1. Van Wieringen, W.N. Lecture Notes on Ridge Regression. arXiv:150909169. 2020 Available online: http://arxiv.org/abs/1509.09169 (accessed on 23 April 2020).
